# Editorial on Special Issue “Recent Advances in Hydrogels for Biomedical Applications”

**DOI:** 10.3390/pharmaceutics16091193

**Published:** 2024-09-11

**Authors:** Marija M. Babić Radić

**Affiliations:** University of Belgrade, Innovation Center of Faculty of Technology and Metallurgy, 11000 Belgrade, Serbia; mbabic@tmf.bg.ac.rs

The significant progress in design and engineering of advanced hydrogels for biomedical applications is fundamentally driven by the increasing complexity and specificity of challenges in the biomedical field. This synergy of sophisticated biomedical requirements and the science of biomaterials accelerates advanced hydrogel development, expanding their potential for applications in various biomedical segments. Hydrogels, as powerful materials with excellent biocompatibility and the ability to mimic the natural extracellular matrix (ECM) as well as to interface with biological environments, provide ideal biophysiological conditions for cellular processes and tissue regeneration. Additionally, their high water content and tunable properties capable of precisely matching the requirements of specific medical applications make them favorable for a wide range of biomedical uses, including delivery of active agents in a controlled, sustained, and targeted manner (contribution 1−4), tissue engineering as bioactive scaffolds that promote cellular processes and health tissue formation by both bioactivity and replicated ECM structures (contribution 3,5,6), targeted therapies with improved efficacy and reduced side effects using stimuli sensitive hydrogels (contribution 2). This Special Issue highlights the current trends in the development of hydrogels for biomedical applications. 

The original contributions in this Special Issue focus on the development of hydrogels as innovative systems for active agent delivery in sustained, targeted (contribution 1), pH-triggered (contribution 2), and simultaneous multi-target (contribution 3) manners for anticancer and bioimaging (contribution 1,2), wound healing (contribution 3), and *Candida albicans* infection (contribution 4) therapies. This Special Issue also includes two reviews providing a comprehensive overview of the hydrogels based on alginate for cell transplantation (contribution 5) and hydrogels for dental tissue engineering (contribution 6).

Hydrogels designed as drug delivery systems for antitumor therapies represent a significant advancement in this field due to their ability to provide release of various anticancer active agents in a controlled and sustained manner, directly at the tumor site, minimizing side and target-off effects. To overcome the limitations of the systemic delivery of anticancer active agents such as systemic toxicity, serious side effects, and frequent drug administration, an innovative platform designed using a thermosensitive, biodegradable hydrogel with functionalized liposomes that provides local and sustained release of MEK inhibitor and doxorubicin (DOX) was proposed by Dr. Effosini Kokkoli et al. (contribution 1). Thermosensitive hydrogels are in the focus of numerous scientific studies as promising biomaterials for anticancer drug delivery systems (DDS) due to their ability for sol–gel transition in response to temperature changes. This unique property allows thermosensitive hydrogels to be easily mixed with anticancer agents and injected in a liquid form at room temperature without the use of surgical implantation, providing sustained delivery of active agents directly to the tumor site. To design the novel anticancer DDS, Dr. Effosini Kokkoli et al. selected a poly(δ-valerolactone-co-lactide)-b-poly(ethylene-glycol)-b-poly(δ-valerolactone-co-lactide) (PVLA-PEG-PVLA) triblock copolymer, which undergoes a gelation by spherical-to-wormlike micelle transition at physiological temperature (37 °C), compared to the other thermosensitive hydrogels that require high temperatures (≥60 °C) or high salt concentration to achieve phase transition (contribution 1). The authors evaluate innovative DDS in vitro by a multi-compartment tumor organoid model confirming increased nanoparticle uptake with a biomimetic peptide (PR_b) and sustained release from the hydrogel, while the testing in preclinical models of E-cadherin-positive invasive ductal carcinoma highlights the potential for clinical translatability of co-delivering iMEK and DOX from the DDS. This innovative platform designed by Dr. Effosini Kokkoli et al. demonstrated localized, sustained and targeted co-delivery of MEK inhibitors to E-cadherin-positive tumors in combination with traditional chemotherapeutics, to be a safe and promising, noninvasive anticancer therapeutic strategy for the first time. The findings obtained in this study highlight the potential of dual therapy with the hydrogel-nanoparicle system as a neo-adjuvant therapy for reducing large breast tumors to a safe size before surgery or as an adjuvant therapy to eliminate remaining cancer cells post-surgery, enhancing the therapeutic index of toxic drugs, reducing systemic toxicity, preventing metastasis, and improving survival outcomes.

Among thermosensitive hydrogels, pH-sensitive hydrogels are another class of stimuli-sensitive biomaterials that are of paramount importance for use in anticancer therapies due to their significant potential for efficacy and highly targeted treatment with minimized side effects. Dr. Keristina Wagdi Amin et al. developed multifunctional hydrogel nanoparticles that can encapsulate both anticancer drugs and imaging contrast agents (contribution 2). To design this innovative DDS, mitomycin C (MMC) as an anticancer drug and rhodamine B (RB) as an imaging contrasting agent were conjugated to the succinated polyvinyl alcohol polymer (PVA-SA). It was found that the hydrogel nanoparticles with 0.5% and 1.5% RB enhanced the RB quantum yield, emission stability, and also extended RB fluorescence lifetime compared to free RB, highlighting their potential for imaging-guided therapy. Proposed innovative DDS were also evaluated for loading and in vitro release of anticancer drug MMC, confirming their high potential for pH-dependent MMC release. These findings represent an interesting approach to developing advanced anticancer therapies by combining hydrogels, anticancer drugs, and imaging contrast agents in a single entity to achieve precise targeted release of active agents with minimized side and target-off effects.

The increasing incidence of various diseases with specific complexities, such as chronic wounds and cancer, highlights the urgent need to approach this global economic and social burden with an alternative healing model that can address all biophysiological requirements arising from the nature of disease with a single treatment approach. In this regard, one of the revolutionary therapeutic approaches to these diseases relies on the development of innovative biomaterials with properties favorable for the design of multi-target therapies. Hydrogels offer a highly promising platform for the design of multi-target therapies due to their biocompatibility and ability to release a variety of active agents, providing biophysiological support to multiple therapeutic targets within a single treatment approach. Dr. Marija Babić Radić et al., inspired by the nature of the wound healing process and the importance of providing simultaneous support at all stages of the wound healing process, proposed the innovative multi-target wound therapy platform utilizing the synergy effect of hydrogel scaffolds that mimic native extracellular matrix (ECM) and multiple active agents with high wound healing potential (contribution 3). These hydrogel scaffolds based on natural polymers gelatin and alginate, reinforced with titanium dioxide (TiO_2_) nanoparticles, and loaded with allantoin, caffeic acid, and quercetin provide not only passive support to cellular processes through replicated ECM structure but also active stimulation of all critical biophysiological actions during wound healing, including inflammation, proliferation, and remodeling. It was found that gelatin/alginate hydrogel scaffolds reinforced with TiO_2_ nanoparticles possess highly porous interconnected morphology, tunable swelling capacity, porosity, and mechanical property through simple modifications in their chemical composition, highlighting their potential for skin tissue regeneration. Evaluating the biocompatibility of the hydrogel scaffolds in vitro on fibroblasts (MRC5 cells) and in vivo (*Caenorhabditis elegans*) in a survival probe test confirms their safe biomedical use, while results of the in vitro release study emphasize their high potential for simultaneous release of allantoin, quercetin, and caffeic acid, indicating that the release pattern could be tuned by altering the scaffold’s chemical composition. Findings obtained in this study highlight the potential of gelatin/alginate-based hydrogel scaffolds reinforced with TiO_2_ nanoparticles and loaded with allantoin, quercetin, and caffeic acid for use in clinical wound healing treatments, providing improved wound healing in a beneficial and non-invasive manner.

The development of hydrogels with antimicrobial activity is a rapidly growing area of research, driven by the urgent need for new strategies for treating and preventing resistant pathogens and complex infections. Dr. Juliana Campos Junqueira et al. proposed a novel strategy for *Candida albicans* therapy based on caffeic acid phenethyl ester (CAPE) as an antifungal agent and gellan gum (GG) as a carrier system (contribution 4). Synthesized CAPE-GG formulations demonstrated strong antifungal activity against *C. albicans* biofilms at CAPE concentrations from 128 to 512 µg/µL, decreasing fungal viability and *C. albicans* protease activity within 12 h, especially at a 1.0% GG. Dr. Juliana Campos Junqueira et al. highlighted that CAPE-GG was not cytotoxic to human keratinocytes and effectively decreased fungal burden and inflammation in a rat model of denture stomatitis, promoting GG as a promising delivery system for CAPE in treating *C. albicans* infections and denture stomatitis. 

This Special Issue was concluded with two reviews providing a comprehensive overview of the recent advances in hydrogels for biomedical applications, particularly for tissue engineering, cell transplantation, and dental tissue engineering (contribution 5,6). Dr. Alireza Kavand et al. analyzed the recent advances in alginate-based hydrogels as potential formulations for cell transplantation, highlighting their efficacy in treating various diseases and injuries in the field of regenerative biomedicine (contribution 5). Various forms of alginate-based biomaterials, including microspheres, hydrogels, scaffolds, fibers, and bioinks, were discussed, as well as their benefits and limitations for regenerative medicine applications. Additionally, a critical evaluation of the innovations in microencapsulation and 3D-printing of alginate bioinks confirms their potential to engineer complex 3D biostructures that closely mimic native tissue and promote the exchange of oxygen as well as vascularization. The focus of the second review is on current advancements in biopolymers and their uses in 3D bioprinting technologies, highlighting progress in dental tissue engineering (contribution 6). Dr. Suhon Kim et al. proposed a comprehensive overview of the innovations in polymer-based dental tissue engineering and 3D printing technologies, as well as critical discussion of the current state-of-the-art, focusing on critical techniques, including polymeric biomaterials and 3D printing with/without cells for dental tissue engineering applications (contribution 6). The review was completed with section that identified the challenges and future perspectives of the dental tissue engineering research field. The authors highlight that future research should focus on integrating functional 3D bioprinting with advanced imaging and autologous tissue implantation to further translate innovative strategies into clinical practice, while understanding the utilization of stem cells, scaffold materials, and bioprinting technologies remains crucial. 

The scientific articles in this Special Issue represent significant breakthroughs that reveal the diverse and interdisciplinary efforts in developing innovative strategies to engineer hydrogels with properties suitable to address various complexities in biomedical applications. We extend our gratitude to all the authors of this Special Issue for contributing their high-quality research. 

